# Systematic Review on HRV Reference Values

**DOI:** 10.3390/jcdd12060214

**Published:** 2025-06-06

**Authors:** Maximillian Brozat, Irina Böckelmann, Stefan Sammito

**Affiliations:** 1 German Air Force Centre of Aerospace Medicine, 51147 Cologne, Germany; 2 Department of Occupational Medicine, Faculty of Medicine, Otto von Guericke University of Magdeburg, 39120 Magdeburg, Germany; irina.boeckelmann@med.ovgu.de

**Keywords:** autonomous nervous system, parasympathetic, sympathetic, heart diseases, norm values

## Abstract

Heart rate variability (HRV) has been established as a measure for the variation in time intervals between successive cardiac actions as a marker of the autonomic nervous system. However, despite many efforts in this field, there are no reference values that are generally accepted. The objective of this systematic review is, therefore, to present an overview of the studies on HRV normal values published to date, with due consideration of any influencing factors. A systematic database query was carried out in PubMed, Scopus, Ovid Medline, and PsychInfo using the search string “((hrv) or (heart rate variability)) and ((reference values) or (reference range) or (normal values))”. Of the 6640 studies yielded by the query, 58 were used for this systematic review. The STARD-HRV procedure was used to assess the quality of the studies. The studies considered date from 1989 to 2022. The number of subjects examined was between 20 and 84,772. The age of the subjects was between 1 day and 99 years. A total of 51 of the studies examined both male and female subjects. In total, 19 studies used long-term measurements, 22 studies used short-term measurements, and 17 studies used intermediate measuring periods. Many different HRV parameters were analyzed, most often traditional time-domain and frequency-domain ones. Nine studies described the subjects as “healthy” without giving more detailed explanations. There are no generally accepted HRV normal values (yet). Some large studies provide values that may be used for orientation purposes. However, further studies are required to collect HRV normal values. It was not possible to merge the results of the studies in terms of a meta-analysis; this would also not be practical since, among other reasons, the consideration of confounders as well as recording and measuring modalities sometimes vary to a large extent and impede the comparability of the studies. Generally, HRV seems to be influenced by various mechanisms and external factors that are still not fully understood. An exploration of these factors will ultimately allow HRV normal values to be obtained in a manner that is generally accepted.

## 1. Introduction

Heart rate variability (HRV) is a measure of the variation in the time intervals between successive normal heartbeats (referred to as NN intervals) and describes the influence of the autonomic nervous system on heart activity. In order to advance the standardization of the HRV parameters and their measurement, a guideline on measuring and using HRV was published by the North American Society of Pacing and Electrophysiology together with the European Society of Cardiology in 1996 [1]. This guideline already pointed out the necessity for further research, in particular, to enable the determination of normal values depending on influencing factors such as age and gender. Intraindividual measurements of HRV can be easily reproduced and compared over time. However, clinical applications—among others, for risk stratification following, for example, acute myocardial infarction, the early detection of autonomous dysregulation, and preventive medical uses in general—will require interindividual comparisons for which no generally accepted reference values exist to date despite further efforts taken [2,3,4].

The specification of reference values that are generally accepted is impeded by the use of various measuring methods and intervals having different significance. For example, in addition to the traditional ECG measurements used to determine the NN intervals, there are also measurements using heart rate monitors and pulse oximeters with a clearly limited precision [5] and different recording durations: short-term (≤five minutes) vs. long-term measurements (24 h in most cases) [1].

Some studies addressed the collection of reference values [1,6,7,8,9,10,11,12,13]. Since there are physiological factors that have been accepted as influencing factors on HRV [14] by now (such as age, gender, and ethnicity), studies on reference values should also consider these factors in addition to the above-mentioned problems of measuring methods and intervals.

The objective of this review is, therefore, to present an overview of the studies on HRV normal values published to date, with due consideration of the influencing factors mentioned.

## 2. Materials and Methods

This systematic review was conducted under consideration of the PRISMA guidelines [15]; the PRISMA checklist can be found in Supplement S1. The search string “((hrv) or (heart rate variability)) and ((reference values) or (reference range) or (normal values))” was applied to the PubMed, Scopus, Ovid Medline, and PsychInfo databases on 2 November 2022. The hits were then transferred to the Citavi 6 writing software (version 6.12, Lumivero/QSR International).

No restrictions were made in advance with regard to the year of publication, the HRV parameters collected, or the HRV measuring duration, but only original studies containing full texts in English or German and examining healthy persons to determine the HRV normal values were included in the review. Studies on animals as well as studies on humans, which, however, showed an underlying disease, were, therefore, excluded. Studies comparing subjects with a specific disease versus a healthy group of subjects were also included. This study was registered with PROSPERO under registration no. CRD42023388947.

**Figure 1:**
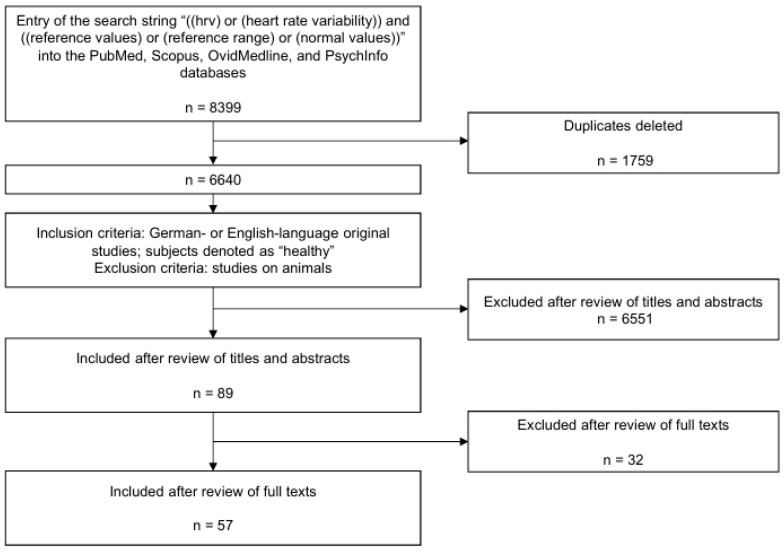
PRISMA flow chart for this systematic review.

The initial search yielded 8399 hits, 6640 of which remained after deleting duplicates. After an independent review of the titles and abstracts by two persons (MB and SS), 6551 of these studies were excluded since they did not meet the inclusion and exclusion criteria. Of the 89 titles remaining, 16 studies lacked a full text, another 12 were excluded in terms of contents of their full texts, and 3 studies were not included for the circumstances described below. The review of the full text was also performed by the two authors above. In the case of a different assessment of the abstracts or the full texts, both reviewers jointly assessed the articles and came to a common judgment. Thus, 57 studies remained that were classified as usable (see Figure 1).

Some of the original studies were excluded due to particularities and are listed here for reasons of completeness. The 2019 study by van den Berg [16] only provided corrected RMSSD (Root Mean Square of successive differences) reference values of female subjects which had previously been incorrectly stated in the 2018 study of the same authors [17]. Therefore, the update is not listed as a separate publication in the table. The studies by Zeng et al. 2014 [13] and Tang et al. 2014 [18] were similar with regard to the patient population, the methods used, and most of the results, so the assumption was made that the study results had been divided into two publications. In order to avoid redundancies, only the study by Tang et al. 2014 [18] was included in the table of results since this study is more extensive (five vs. three tables of results) and was published more recently. For the study by Sammito et al. 2016 [2] listed in Supplementary Material Table S1, a revised table was published in 2017 due to a methodological error. Apart from that, no changes were made. Therefore, the 2016 study [2] was included for the methodological information, but the reference values were taken from the 2017 correction [12].

In order to systematically verify the quality of the studies listed in this study, we used the checklist proposed in the STARD Guidelines of 2015 [19] and modified it for studies on HRV [20]. In accordance with this checklist, a maximum of 25 points can be achieved if all the criteria are met. Item 5 “Study uses within-subject design” was changed to “Study uses between-subject design” in order to apply this list in a purposeful manner to studies that collect reference values of HRV. The score achieved by the individual studies is listed in Table S1.

## 3. Results

A total of 57 original studies were found that addressed the collection of normal values of HRV [2,8,9,11,17,18,21,22,23,24,25,26,27,28,29,30,31,32,33,34,35,36,37,38,39,40,41,42,43,44,45,46,47,48,49,50,51,52,53,54,55,56,57,58,59,60,61,62,63,64,65,66,67,68,69,70,71]. The studies date from 1989 [24] to 2022 [28,46]. Due to the inclusion criteria of this review, all the original studies considered in this review examined healthy subjects or subjects denoted as “healthy”.

The size of the patient populations of the original studies ranged from 20 [24] to 84,772 subjects [66]. Twelve studies examined less than 100 subjects, 32 studies examined 100 to 1000 subjects, and 14 studies conducted HRV measurements on more than 1000 subjects. Of the 32 studies with a patient population of 100 to 1000 subjects, 25 studies examined less than 500, and 7 studies more than 500 individuals.

The age range of the subjects of all the studies covers 1 day [52] to 99 years [68]. In 51 studies, measurements were conducted on male and female subjects, and/or results were listed separately; 7 studies [21,24,26,33,35,44,71] did not differentiate the results by gender or examined one gender only.

Various ECG systems were used to conduct the HRV measurements. Eleven original studies, however, only stated that an ECG unit had been used as the measuring method, but did not specify the manufacturer or the model used. The study by Otsuka [71] does not include any information as to whether the data were collected by means of an ECG system and thus does not mention a manufacturer or model designation.

The sampling rates of the ECG recordings of the original studies were between 32 Hz [69] and 1000 Hz (e.g., [11,53]). Van den Berg even varied the sampling rate of his measurements from 500 Hz to 1200 Hz [17]. A total of 23 studies did not provide information on the sampling rates used (e.g., [41,43]); 11 studies used 1000 Hz (e.g., [2,36]). The remaining 25 studies used sampling rates lower than 1000 Hz.

The measuring and recording periods of the studies were also inconsistent. In 19 studies, long-term measurements of 24 h were conducted, and in 22 studies, short-term measurements were carried out with recording periods varying between 10 seconds [17,66] and 5 minutes (among others, [23,29]). However, there were also intermediate measuring periods: Massin et al. [51] used 400 min recordings; Goto et al. [39] evaluated intervals of 5 hours. In total, 17 of the 58 studies did not select a traditional long-term measuring period of 24 h or a short-term measuring period ≤ 5 minutes for their calculations. The exact time of day of the recording, which is of interest in the short-term measurements, was described by 10 of the 22 studies with a measuring period of ≤five minutes. The range of the time intervals chosen to describe the exact time of day at which the measurements were taken varied between five minutes [11] and nine hours [53]. Three studies described a period of the day without specifying the exact times of day [24,49,69], while another eight studies did not indicate any time of measuring (among others, [30,55]).

With regard to the HRV parameters analyzed in the studies, it was found that some of them were used more frequently. The traditional time-dependent parameters were collected frequently: RMSSD in 46 studies, SDNN (Standard deviation of NN intervals) in 41 studies, and pNN50 in 28 studies. Among the frequency-dependent parameters, the following ones were analyzed most frequently: LF (Low frequency) (in 43 studies), HF (High frequency) (in 42 studies), and an LF/HF ratio (in 31 studies). Nonlinear parameters were considered in a smaller number of studies: DFA1 in six, DFA2 in five, and SD1 and SD2 (Even though SD1 and SD2 are counted as time-related HRV parameters according to Sassi et al. [3], we have listed them here as nonlinear parameters, as these are classified as such in the underlying studies. Formally, however, SD1 and SD2 should be counted as time-related HRV parameters.) in three studies each. Furthermore, several studies analyzed parameters that are used less frequently, and also calculated new parameters. Besides the “traditional” HRV parameters listed above, a total of 50 of the 58 original studies provided further calculations. In their study published in 2012, Voss et al. [9] analyzed numerous parameters such as the entropy parameters renyi4, a21rr, and wsdvar, which were not examined in any other of the studies considered. In the 1999 study by Otsuka et al. [71], the frequency-dependent parameters were extended and/or substituted by “quadrisecundan” for HF and “decisecundan/quadrisecundan” for LF/HF.

Influencing factors (“confounders”) were considered in most but not all the original studies. In 9 of the 58 studies, the designation of the subjects as “healthy” was the only inclusion and/or exclusion criterion. The remaining 49 studies used numerous criteria for this purpose, some of which depended on the age or the place of work of the subjects. Baldzer et al. [24] referred to the birth weight and the APGAR scores of the subjects since their cohort consisted of neonates. Bonnemeier et al. [27] excluded shift workers from their measurements. Various other exclusion criteria were determined in order to sort out known confounders of HRV and HRV analyses in particular. This included, among others, cardiovascular diseases (cf. [38]), neurological diseases (cf. [41]), diabetes mellitus (cf. [45]), the use of medication (cf. [31]), and cardiac arrhythmias (cf. [50]) as well as others.

The reference values collected in the original studies were most frequently presented in relation to the gender and age of the subjects. In 49 studies, the HRV parameters were presented depending on age, in 42 studies depending on gender, and in 32 studies in relation to other variables such as the body mass index (cf. [70]) or the heart rate (cf. [67]). Only three studies did not present a connection to the age or gender of the subjects. Farah et al. did not relate HRV to any variables [33], Kobayashi et al. selected the salivary alpha-amylase as a reference value [44], and Mehta et al. used the heart rate of the subjects [52].

The 58 original studies described above presented the results mainly in the form of the mean value ± standard deviation (SD) or percentiles. A total of 20 studies only presented the mean value ± SD (e.g., [55,60]); another 20 studies described the mean value ± SD and percentiles in addition (e.g., [48,53]). Seven studies only presented percentiles (e.g., [11]). Deviating from these approaches, the remaining eleven studies used, for example, only the mean value ([24]) or described a lower and an upper limit value [22] without providing any further explanation. For some HRV parameters (especially LF and HF power), it should be considered that mean ± SD were sometimes calculated from raw powers and sometimes from log powers. These values are not easily comparable [72].

The wide range of analysis periods used and the HRV parameters included, as well as the different presentations, make it difficult to perform an analysis to combine the different results, such as a meta-analysis. Table 1 shows an example of the studies that presented mean values and standard deviation for the HRV parameter RMSSD for an analysis period of 5 min. Of the total of twelve studies that analyzed RMSSD over 5 min, this could only be obtained from the published results in four studies [8,23,30,55], four further studies presented the results as percentiles [11,28,29,61], one study as quartiles [36], and in three further studies the median was also presented in addition to the percentiles [9,53,63].

The score achieved in accordance with the STARD criteria was between 9 points [66] and 23 points [36].

A summary of all the studies is listed in Table S1.

## 4. Discussion

In the course of this systematic review of the literature, all the studies on HRV normal values found were collected, analyzed, and categorized in tabular form. Altogether, the studies considered were found to be highly heterogeneous. In some cases, the populations and origins of the patients, age ranges, HRV parameters analyzed, measuring and evaluation instruments used, and confounders vary considerably among the studies found. The broadest consensus seems to exist with regard to the time frames of the examinations here, where the distinction between short-term and long-term measurements is predominant, although there are some studies investigating intermediate durations.

While the known circadian rhythm of HRV can be largely ignored in long-term measurements over 24 h [26,73], this is not true for short-term measurements. Accordingly, the latter strongly depends on the time of recording. In the available studies on HRV reference values with short-term measurements, the times of recording in the course of the day differed significantly in some cases. For studies that collect reference values for HRV short-term measurements, it is, therefore, crucial that the own measurements are conducted at a similar time of day since, otherwise, a comparison is not possible simply due to circadian fluctuations. Longer recording periods of up to nine hours [53] do not take sufficient account of this fact.

An important characteristic of the quality of studies on HRV normal values is the consideration of the influencing factors that can change HRV [4]. HRV can be significantly affected by factors such as age, gender and ethnic origin, numerous diseases, and the use of medication. Although age was considered as a confounder in 49 studies and gender in 42 studies, other influencing factors were considered in only 32 studies. However, a more accurate and detailed description of influencing factors such as the origin, mental and physical impairment, and medication used is important because they can cause a change in HRV. Many of the studies already used criteria to include only “healthy” subjects. These criteria often comprise known confounders such as diabetes mellitus, the use of medication, psychiatric disorders, or pregnancy. Furthermore, “cardiovascular and neurological diseases” were often excluded, although this general wording does not indicate how these conditions were determined. In most cases, the studies mentioned the history of the subjects and a physical examination so that potentially relevant diseases were ruled out by indirect methods, for example, by using the fact that the subjects did not take medication or had a resting ECG within normal limits as a measure of health. Active exclusion of diseases and conditions that affect HRV is usually much more complex. This complexity must be weighed against practicability and cohort size. However, since the role of confounders is increasingly understood and examined, they must be taken into account accordingly. Future studies should, therefore, consider diseases and influencing factors that affect HRV by interviewing and/or examining subjects accordingly, and exclusion should be made if such diseases and factors are present. A self-assessment of the subjects as “healthy” does not suffice.

There is a category of confounders that may be underrepresented in the studies from our point of view, namely lifestyle factors, which may also have an impact on HRV [74,75,76]. Consuming alcohol and smoking cigarettes in the hours or days before or during the ECG recordings are indeed often used as exclusion criteria. However, other lifestyle factors such as regular exercise or sports and a more detailed description of the frequency are rarely found in the studies, although the use of HRV for training and recreation has been playing a role for quite some time not only in top-level sports [77], but also—due to commercial smartwatches—within the hobby and amateur sector [78]. This applies equally to other lifestyle factors, such as sleep behavior, increased body fat percentage or an increased body mass index, and stress [14] but these were also only rarely or not at all taken into account in the studies listed. When collecting normal values, it should, of course, be avoided to create a subpopulation of physically active or inactive persons, as regular physical and/or sporting activity has an influence on HRV [14]. This can be done, for example, by simultaneously recording physical activity over the survey period or by using validated questionnaires such as the International Physical Activity Questionnaire (IPAQ) [7] or other questionnaires. However, a more detailed analysis of the lifestyle factors—physical activities in particular—could be taken into consideration in future studies on HRV normal values and can be easily implemented by asking the subjects in a questionnaire.

As described in the Section 3, most of the studies present the HRV results in the form of a mean value and an SD with and without additional percentiles. Both forms of presentation are, thus, widely used and established. Percentiles have the advantage that outliers are not only described statistically by the SD, but can also be assessed in relation to the overall distribution of the HRV values. Mean value and SD should only be reported if the distribution of values is (more or less) Gaussian. If the distribution is non-Gaussian, then percentiles better reflect the distribution of values. Furthermore, it should always be noted that the advantage of the direct readability of the distribution from percentile curves is offset by the fact that it is more difficult to make further calculations with percentile data. For HRV normal values, this might have the benefit that larger groups of outliers—which would need to be analyzed with regard to their cause—are more easily identified and not “lost” in the SD.

The classification into time-domain, frequency-domain, and nonlinear parameters has become widely accepted. The nonlinear parameters in particular, which deviate from the “traditional” parameters, have been significantly extended and added as new HRV parameters. However, a decisive breakthrough in HRV measurement and clinical application by means of new nonlinear parameters has not (yet) been achieved, even though there are some promising approaches [3]. The large number of different HRV parameters that have also been used across the available studies to determine HRV parameters impedes the comparability of results since many of the included studies only use a small number of HRV parameters. However, an application of the same HRV parameters in different studies would have the advantage that they become comparable and can be scientifically consolidated by means of meta-analyses as appropriate. For this reason, future studies that calculate the HRV normal values should focus on the traditional parameters while previously rare or fully unknown parameters could be included as a useful addition.

This systematic review has its strengths and weaknesses. One strength of this review is that the selection of the search criteria and the inclusion of four scientific databases produced a large number of hits, which were then systematically analyzed. This allowed a large number of potentially relevant studies to be captured. It does not rule out, however, that individual studies were not found since they were listed in other databases, did not meet the selected inclusion and exclusion criteria, or were eliminated by mistake. The probability that studies were excluded by mistake was reduced by the independent screening of the database hits by the two authors.

Another strength of this study is that the original studies found were examined for their methodological quality. For example, this systematic review incorporated details as to whether and how influencing factors were taken into account, what kinds of subject groups were covered, and whether the measurements were of the short-term or the long-term type.

What constitutes a limitation of the systematic review is the restriction to English- and German-language original studies. This issue must be taken into consideration when evaluating this study. However, it must also be taken into account that the major scientific journals publish in the English language, so ruling out other languages rather meant ruling out national journals. It cannot be excluded that this may have resulted in individual studies not being included in this study, but the authors estimate the resulting bias to be low.

Another weakness of this study is that only a list of the studies could be prepared. This approach was chosen because it is neither possible nor purposeful to consolidate the results of the listed original studies on reference values that are generally applicable. HRV is affected by various influencing factors and confounders that were not taken into account in most of the studies available. Other confounders such as ethnic origin, age, gender, and the health condition of the subjects also have an impact on HRV. Due to the high degree of heterogeneity of the studies found, it would not have been possible to consolidate the results without not taking these issues into account. This was why we refrained from a consolidation.

The present review shows that it will be fundamentally important that future studies that want to determine reference values for HRV should be based, among other things, on a common analysis period, influencing factors to be taken into account, usable measurement technologies, and an agreement on which HRV parameters should be analyzed and which statistical methods should be used. To this end, a corresponding task force might first have to define these factors on which future studies could be based. The difficulties shown here with the analysis of only one HRV parameter in the sense of a summary of all reference values show how important such an approach would be. In summary, it can be said that—despite repeated efforts [1,3]—it has not yet been possible to achieve a harmonization of the HRV analysis and collect normal values that are generally applicable. However, individual studies with large sample sizes are suitable already today to close at least parts of this gap. However, it would still be necessary to extend this knowledge.

## Figures and Tables

**Table 1:** Overview of studies in which reference values for RMSSD with mean and standard deviation (SD) were listed.

Study	Group	*n*	Mean ± SD
Agelink et al., 2001 [23]	Male, 17–25 years	58	1.65 ± 0.32
	Female, 17–25 years		1.58 ± 0.32
	Male, 26–35 years	123	1.58 ± 0.22
	Female, 26–35 years		1.54 ± 0.30
	Male, 36–45 years	47	1.45 ± 0.30
	Female, 36–45 years		1.35 ± 0.23
	Male, 46–55 years	42	1.31 ± 0.21
	Female, 46–55 years		1.28 ± 0.22
	Male, 55+ years	39	1.25 ± 0.21
	Female, 55+ years		1.33 ± 0.27
Dantas et al., 2018 [30]	35–44 years	982	34.9 ± 17.5
	45–55 years	1252	30.0 ± 15.6
	55–64 years	536	26.7 ± 15.3
	65–75 years	104	27.7 ± 23.6
Kim et al., 2011 [8]		3408	29.7 ± 18.1
Park et al., 2007 [55]		637	27.3 ± 15.6

## Data Availability

No new data were created or analyzed in this study.

## Supplementary Materials

The following supporting information can be downloaded at https://www.mdpi.com/article/10.3390/jcdd12060214/s1, Supplement S1: PRISMA Checklist. Table S1. Results of the systematic review.

## Abbreviations

The following abbreviations are used in this manuscript:

HRV	heart rate variability
SD	standard deviation
RMSSD	Root Mean Square of successive differences
SDNN	Standard deviation of NN intervals
LF	Low frequency
HF	High frequency
