# Supplementary material for: Systematic Review on HRV Reference Values
Source: J Cardiovasc Dev Dis. 2025 Jun 6;12(6):214. doi: 10.3390/jcdd12060214 (PMC12194801; doi:10.3390/jcdd12060214)
Supplement: Supplementary file 1 [file jcdd-12-00214-s001.zip › eTable S1.pdf]

Journal of
Cardiovascular Development and Disease

MDPI

Table S1. Results of the systematic review

Study	Cohort	n	Age [years]	Measurement interval, time of day if short-time measurement	HRV parameters	Device	Confounders considered	References values presented for	Sampling rate in Hz	Norm values presented as…	STARD-HRV
Acharya et al. 2004	Chinese healthy population	150 (75 m, 75 f)	5 - 70	20 minutes, no description of the exact time of day	SDNN, SDSD, RMSSD, pNN50, Triangle index, TINN; LF/HF; SD1/SD2, ApEn, LLE, α s; α L	ECG (PowerLab/ 16SP system (ADI instru-ment)	n/a	age groups	400	upper/ lower limit without more precise specification	12
Agelink et al. 2001	healthy subjects	309 (151 m, 158 f)	18 - 77	5-minute resting HRV between 9-11.30am	CVRR, RMSSD, VLF power, TP, VLF, LF, HF	ECG	CVD, endocrinologic, neurologic, psychiatric disorders, alcoholism, polytoxemia	age & gender groups	n/a	mean ± SD	21
Baldzer et al. 1989	full-term infants	20	30 - 60 h post-natal	continuous recording of at least 4 mins during sleep values for all stable 100-s segments of recording available for each infant"	TP, LF*, RSA power, %LF*, %RSA, LF/RSA	neonatal heart rate and respiration monitor and four channel tape recorder	birth weight 2800-3800 g; APGAR-scores >7 at 1 and 5 minutes, no congenital anomalies, no maternal drug exposure, no chronic/ acute fetal distress	gestational age, postnatal age, birth weight; infants were divided into two groups according to RSA power and LF/RSA ratios	n/a	mean	17
Beckers et al. 2006	healthy subjects	276 (141 m, 135 f)	18 - 71	24h-recordings split into daytime and nighttime	meanRR, SDNN, RMMSD, pNN50, TP, LF, %LF, HF, %HF, LF/HF, 1/f slope, FD (fractal dimension), ApEn, DFA1, DFA2, CD, %CD difference, S value, LLE	Holter ECG (ELA Medical Holter monitoring)	normal ECG and physiological examination, detailed medical history with no history of DM, hypertension, CVD, neurological and/or psychiatric diseases	age, gender, day or night time, heart rate	200	mean ± SD	17
Bilan et al. 2005	healthy subjects	48	35 - 55 (m 40 ± 6; f 38 ± 8)	24h ambulatory 3-lead ECG monitoring; night period midnight-6am, morning period 6am-10am, day period 3pm-8pm	LF, HF, LF/HF	Holter ECG (Oxford Medilog FD3 digital recorders)	no significant findings in medical history, no abnormalities in physical examination and resting ECG, arterial BP, resting echocardiography, laboratory tests (blood cell count, serum total cholesterol, fasting glucose, electrolytes). Excluded: low technical quality ECG, >10 ectopic beats per hour, conductibility disorders, ST deviations, <270 consecutive 5 min intervals	age, gender, timepoint	n/a	mean ± SD	15

Study	Cohort	n	Age [years]	Measurement interval, time of day if short-time measurement	HRV parameters	Device	Confounders considered	References values presented for	Sampling rate in Hz	Norm values presented as…	STARD-HRV
Bonnemeier et al. 2003	healthy volunteers	166 (85 m, 81 f)	20 – 70 (42 ± 15)	24h ambulatory ECG, two-channel time tracking Holter recorders Holter recording initiated between 9am-10am; waking at 7am, 6-8 hours of sleep, meal eating advised around 8am, 1pm, 7pm	RMSSD, SDNN, SDNNi, SDANN, NN50, geometric triangular index TI	Holter-ECG (Tracker II, Reynolds, Hertford, UK)	no evidence of organic cardiac disease as determined by medical history, physical examination, 12-lead ECG, echocardiography, no DM, hypertension, overweight (BMI >25 kg/m^2), neurologic and psychiatric diseases; no shift workers; no chronic medication except oral contraceptives	age, gender, timepoint	n/a	mean ± SD	18
Brinth et al. 2022	subset of a large Danish population-based study	6891 (3152 m, 3739 f)	18 - 72	5 min supine rest, thereafter 7 min measurements in supine rest and normal breathing; the last 5 min of the 7 min chosen to be analyzed. All examinations between 8am-3pm, lasting about 90 min. At least 6h of fasting before, asked to abstain from smoking at least 1h prior to the examination.	meanHR, meanRR, STDHR, STDNN (standard deviation of instantaneous of HR/NN values), RMSSD, pNN50, VLF, LF, LFnu, HF, HFnu, LF/HF	'E-motion' heart rate monitor device	Exclusion: not being born in Denmark, not being a Danish citizen, or being pregnant	age, gender	250	percentiles (5th, 25th, 50th, 75th, 95th)	22
Christensen MMB et al. 2021	Greenlandic Inuit from the Greenlandic population study 2018 and the town Qasigiannguit 2020	472 (181 m, 291 f)	20 - 80 (53.9 ± 13.1)	5 min after 5 min supine rest, no description of the exact time of day	SDNN, RMSSD, LF, HF, TP	ECG Handheld device Vagus (Medicus Engineering, Aarhus, Denmark)	DM	age, gender	n/a	5th percentile, 95% KI	18
Dantas et al. 2018	Non-medicated, healthy participants of the Brasilian Longitudinal Study of adult health	2874 (1680 m, 1194 f)	35 - 74 (median 47, IQR 11)	5 minutes, supine position, quiet and temperature-controlled room (20-24 °C)	N-N-variance, SDNN, pNN50, RMSSD, VLF, LF, HF, LF/HF, Ln LF, Ln HF	ECG (ECG Micromed, Brazil)	not pregnant, no medication except oral contraceptives and vitamins, no anamnestic CVD, cerebrovascular disease or cardiac surgery or vascular stents, no obesity, no DM, no hypertension, no chronic kidney disease, no common mental disorders	age, gender	250	mean ± SD and percentile (2.5th, 10th, 25th, 50th, 75th, 90th, 97.5th)	20

Study	Cohort	n	Age [years]	Measurement interval, time of day if short-time measurement	HRV parameters	Device	Confounders considered	References values presented for	Sampling rate in Hz	Norm values presented as…	STARD-HRV
Dietrich et al. 2006	swiss healthy subjects	499	50 - 70	three-lead 24h ECG, mean duration 22.3h ± 2.1h	TP, LF/HF	ECG (Aria, Del Mar Medical Systems, Irvine, CA, USA)	excluded: general or spinal anaesthesia 8 days prior to ambulatory ECG, myocardial infarction 3 months prior, taking digitalis, atrial fibrillation, recording time <18h, insufficient recording quality; inclusion criteria: Non-smoking, no history of CVD, high BP, DM, free of medications	age, gender	Frequency response 0.05-40 Hz, resolution 128 samples/s	percentile curves (5th, 25th, 50th, 75th, 95th)	20
Ergün 2008	Healthy elder people living in nursing homes in Ankara, Turkey	38 (24 m, 14 f)	m 65 - 88, f 62 - 86,	1h recorded in resting supine position after 15 minutes of rest at standard room temperature, no description of the exact time of day	TP, VLF*, LF*, HF*, LF/HF*, nHF*, nLF* (n HF: Normalized HF = HF/TP—VLF × 100; n LF: Normalized LF = LF/TP—VLF × 100)	ECG (PM4 Graseby Medical, UK and a custom-made triggering device generating a TTL (transistor transistor logic) puls)	no CVD, pulmonary disease, hypertension, endocrine abnormalities, cerebrovascular disease, Parkinson´s disease, dementia, or any medication that could cause cardiac rhythm changes	age, gender	1000	mean ± SD	17
Farah et al. 2014	Brazilian high school students	1152 m	14 - 19 (16.6 ± 1.2)	About 30 minutes rest, then 10 minutes in supine position	meanRR, SDNN, RMSSD, pNN50, variance, LF, LFnu, HF, HFnu, LF/HF	Wearable heart rate monitor (Polar RS 800CX; Polar Electro Oy Inc, Kempele, Finland)	excluded: known DM, CVD, neurological or mental disabilities; consumption of caffeinated beverages 12 hours prior to the HRV evaluation; use of alcohol, tobacco, illicit drugs, any physical exercise training 24h prior	Influence testing of waist circumference, BMI, systolic BP, diastolic BP, physical activity by means of regression coefficient; age	n/a	mean ± SD and percentiles (1st, 5th, 10th, 25th, 50th, 75th, 90th, 95th, 99th)	16
Faulkner et al. 2003	healthy adolescent volunteers from USA	70 (26 m, 44 f)	13 - 18 (15.0 ± 1.6)	24h ambulatory ECG monitoring with short-term evoked cardiovascular measures	SDNN, SDANN, SDNN-Index, RMSSD, pNN50, TP*, LF, HF	ECG Holter (Marquette Series 8500 Holter recording system (Marquette Electronics, Milwaukee, WI))	excluded: acute or chronic illness, pharmacological maintenance therapy for asthma resulting from the potential adrenergic effects of bronchodilators; no food or beverage consumption, smoking 30 mins before testing, caffeine intake and smoking 24h prior was unrestricted	age, gender, ethnicity	128	mean ± SD	16
Finley 1995	normal subjects	61	1 month – 24 years	24h tape recordings in the home setting	LF*, HF*, LF/HF*, TP*	ECG	normal resting ECG	age, awake, active sleep, quiet sleep	500	mean ± SD	14
Gasior et al. 2018	healthy children of caucasian ethnicity	312 (153 m, 159 f)	6 - 13	5 minutes during regular school day between 8am and 2pm, supine position; 5 minutes rest in supine position before the examination	SDNN, RMSSD, pNN50, VLF, LF, HF*, TP1, TP2, LF/HF*, nLF, nHF	12-channel ECG (Custo cardio 100 12-channel PC ECG system; Custo med GmbH, Ottobrunn, Germany)	included: 6-13 years, absence of diseases and/or regular use of medications affecting the cardiopulmonary and/or interfering with the nervous system, and not being an active athlete in any sport	age, gender, heart rate, respiratory rate, BMI	1000	Quartile	23

Study	Cohort	n	Age [years]	Measurement interval, time of day if short-time measurement	HRV parameters	Device	Confounders considered	References values presented for	Sampling rate in Hz	Norm values presented as…	STARD-HRV
Geovanini et al. 2020	healthy subgroup of the Baependi Heart Study	543 (225 m, 318 f)	18 - 60 (40 ± 14)	3-channel 24h Holter ECG during daily activities	SDNN, SDANN, RMSSD, pNN50,	ECG Holter (Cardiolight, CardioSmart Office CS-530-CARDIOS))	excluded through self-reporting: hypertension, DM, stroke, cancer, myocardial infarction, cardiac revascularization surgery, angioplasty, those receiving a medication for hypertension, DM, dyslipidemia, those taking a beta-blocker, current smokers, and obese subjects (BMI ≥30 kg/m^2), atrial fibrillation/atrial flutter, pacemaker	age, gender	1000	mean ± SD and percentile (5th, 25th, 50th, 75th, 95th)	20
Gerritsen et al. 2003	healthy caucasian subjects	191 (98 m, 93 f)	50 - 75 (m 62.0 ± 7.4, f 63.3 ± 7.4)	3 min, between 8.30am and 4pm, supine rest, room temperature 19-22 °C	meanRR, SDNN, LF*, HF*, LF/(LF+HF)	bipolar ECG chest-lead	no anamnestic neurological, cardiovascular disease, COPD, hypertension or antihypertensive medication	age, gender	1000	mean ± SD and percentile (10th, 90th)	18
Goto 1997	healthy japanese children,	60 (25 m, 35 f)	3 - 15	5h examination (0-5am) on a day with normal daily activity and sleep pattern	meanRR, SDNN, SDANN, RMSSD, pNN50, LF, HF, LF/HF	ECG (no precise description of the device) Holter analysis via DMW-9000H scanner (Fukuda Denshi, Tokyo, Japan))	organic disease excluded by detailed medical histories and physical and cardial examinations, including two-dimensional echocardiography and conventional electrocardiography	age, gender	125	mean ± SD	15
Harteveld et al. 2021	subjects from Netherland, England, Germany, Switzerland, Spain, Greece, Hungary	4820	0.5 - 25	several measurements from 2 min up to 7 min	heart period, PEP (pre-ejection period; time between ventricular depolarization and and start of left ventricular outflow; shorter PEP is associated with increased SNS activity), RSA (measure of PNS activity), RMSSD, SDNN	ambulatory impedance cardiography and ECG (5fs VU Ambulatory Monitoring system, VU University, Amsterdam, Netherlands)	combined data of participants of five different cohorts with different study protocols: NTR (Netherlands twin register), FemNAT-CD (Neurobiology and Treatment of Adolescent Female Conduct Disorder), MINDS (Mother-Infant Neurodevelopment Study), Nederend et al., The ABCD (Amsterdam Born Children and their Development) study	age, gender	ECG: 1000; ICG 250	mean ± SD and percentile (2.5th und 97.5th)	18
Irshad et al. 2018	patients from Pakistan	32 (23 m, 14 f)	36 ± 15	12-channel ECG performed in each case, 10 heart cycles to assess heart rate; 24 hours after the operation	SDNN, SDANN, RMSSD	ECG (CF card life, Holter ECG, Reynolds medical)	patients with healthy ECG, detailed medical history taken; cardiovascular, psychiatric, neurological disorders, DM excluded	ethnicity	n/a	mean ± SD	12

Study	Cohort	n	Age [years]	Measurement interval, time of day if short-time measurement	HRV parameters	Device	Confounders considered	References values presented for	Sampling rate in Hz	Norm values presented as…	STARD-HRV
Jarrin et al. 2015	children from the Québec Longitudinal Study of Child Development	1036 (481 m, 555 f)	10.2 ± 0.3	majority of recordings between 8-11am; 20 minutes resting, then 1 hour measurement (SD 21 min)	SDNN, SDANN, SDNNi, pNN50, RMSSD, SDANN, VLF, LF, HF, LF/HF	ECG (8500 Marquette MARS Holter monitor; GE Marquette Medical Systems, Milwaukee, Wisconsin, USA)	cardiovascular pathology excluded by ECG reviewing of a board-certified pathologist (PP)	age, gender, heart rate	3	mean ± SD and percentile (5th, 25th, 50th, 85th, 95th)	21
Jensen-Urstad et al. 1997	healthy individuals	101 (49 m, 52 f)	20 - 69	24h	SDNN, SDANN, SDNNi, RMSSD, pNN50, TP*, VLF*, LF, HF, LF/HF	2-channel ECG (Reynolds Sherpa, Reynolds Medical, Hertford, England)	non-smokers, no medication, no history of neurological disorders or cardiac disease, no family history of early death in cardiovascular disease; sedentary or moderately physically active, normotensive, normal resting ECG, subjects >40 years, normal maximal exercise test and normal echocardiogram at least 18h had to be analysable to be included and not more than 4% of non-normal RR intervals	age, gender, time (day/night, day 7.30am - 9.30pm, night 0am - 5am)	n/a	mean ± SD	16
Kim et al. 2011	healthy Korean participants	3396 (2679 m, 717 f)	18 - 65	8-12am; comfortably seated, electrodes on wrists and left foot, 5 minutes measurement	SDNN, RMSSD, TP, LF, HF, LF/HF	ECG (SA-2000E model, Medi-core, Seoul, Korea)	psychiatric disorders assessed through Mini-international-neuropsychiatric interview (MINI), abnormally high SDNN	age, gender, (smoking, acohol consumption, coffee consumption, exercising; BMI, heart rate, BP)	n/a	mean ± SD and percentile (10th, 25th, 50th, 75th, 90th)	15
Kobayashi et al. 2012	healthy young Japanese men/ students	417 m	20 - 29	6.30-7.30am; 1 minute sitting rest, then 2 minutes of recording; repeated on the next day to confirm intra-individual reproducibility	HP (Heart period), SDNN, RMSSD, CVRR, lnLF, lnHF, ln(LF/HF), Skewness, Kurtosis	portable heart rate monitors (AC301; GMS, Japan)	n/a	Salivary Alpha-amylase	1000	mean ± SD and percentile (5th und 95th)	18

Study	Cohort	n	Age [years]	Measurement interval, time of day if short-time measurement	HRV parameters	Device	Confounders considered	References values presented for	Sampling rate in Hz	Norm values presented as…	STARD-HRV
Koskinen et al. 2009	healthy young Finnish adults	1780 (831 m, 949 f)	24 - 39	single channel chest lead ECG; After at least 15 minutes of comfortable rest in supine position during ultrasound studies, 3 minutes of metronome controlled breathing at 0.25 Hz, these 3 minutes used to compute the time- and frequency domain HRV indices; different time groups: 7-8 am, 8-9am, … 12-13pm, after 13pm	SDNN, RMSSD, TP, LF, HF, LF/HF	ECG	excluded: three or more ectopic beats, problems in data recording or saving, hypertension with and without medication, lipid lowering, antidepressive or antipsychotic medications, DM or impaired fasting glucose, pregnant women, breast-feeding women	age, gender, (heart rate, BMI, mean arterial pressure)	200	mean ± SD and 95%-reference limits	19
Latorre-Roman et al. 2022	healthy children from rural and non-rural schools in Andalusia, southern Spain	167 (88 m, 79 f)	3 - 6 (4.68 ± 1.21)	10 minutes, sitting, breathing spontaneously	RMSSD, SDNN, HF, LF, LF/HF	1-channel ECG (Firstbeat Bodyguard 2 (Firstbeat Technologies Ltd., Jyväskylä, Finland)	excluded: chronic diseases, including metabolic or endocrine disorders, DM, asthma, and any condition interfering with physical exercise	age, gender	1000	mean ± SD	15
Lee et al. 2018	consecutive patients	1828 (1185 m, 643 f)	20 - 84	10 minutes in the morning and after an overnight fasting, subjects lying quietly, normal breathing, quiet environment	SDNN, RMSSD, LF, HF, ShanEn, ApEn, SampEn, DFA1, DFA2	ECG	no serious comorbid conditions, self-reportedly no active disease or ongoing medications that can potentially disturb the autonomic cardiac function	age, gender	1000	mean ± SD	21
Longin et al. 2005	healthy neonates	80 (45 m, 35 f)	24 - 168 h	10 minutes, at room temperature, in the morning, 0.5-1h after feeding; resting/lying position	SDNN, RMSSD, pNN50, pNN25, LF*, MF*, HF*	1-channel ECG (VAGUS 2100, Sigma-Medical-Engineering, Thum, Germany)	birth weight at least 2400g, no medication, no cardiological or neurological abnormalities, APGAR 1 min >4, APGAR 5 min >7; no mothers who had used any medication during pregnancy with possible effects on the cardiovascular system, or with acute or chronic diseases as well as drug abuse and smoking	age	256	mean ± SD and percentile (5th und 95th)	21

Study	Cohort	n	Age [years]	Measurement interval, time of day if short-time measurement	HRV parameters	Device	Confounders considered	References values presented for	Sampling rate in Hz	Norm values presented as…	STARD-HRV
Longin et al. 2009	healthy children from schools in Mannheim, Germany	100 (51 m, 49 f)	6 - 15	baseline: every 2 min during a 5 min period; quiet room, in the morning, 23-25 °C, no drinking or eating 2h before testing; rhythmic breathing test; Tilt-table test; Valsalva manoeuvre	SDNN, VC (coefficient of variation), RMSSD, LF, MF, HF, TP	1-channel ECG, bedside monitor (SUEmpathy 100; Suess/ Sigma -Medical - Engineering, Aue, Germany)	exclusion: age<6 years (compliance), presence of disease compromising the central ANS (e.g., bronchial asthma, epilepsy, migraine, DM), any medication; physical and neurologic examination before testing	age group (6-11 years, 12-15 years) (gender: no significant differences found)	256	mean ± SD, median and percentile (5th, 25th, 75th, 95th)	19
Massin et al. 1997	infants and children	210 (102 m, 108 f)	3 days to 14 years	24h, atleast 23h recorded to be accepted	SDNN, SDNNi, SDANNi, RMSSD, pNN50, VLF*, LF*, HF*, LF/HF*	2 channel ECG (MR45 Oxford recorder)	included: sinus rythm, normal medical history and examination, no tobacco use, no medications including estrogen	age	n/a	mean ± SD	15
Massin et al. 2001	healthy infants	587 (286 m, 301 f)	5 - 26 weeks	400 minutes, overnight sleep monitoring from 6pm until 8am, normally dressed, room temperature 20 °C, usual sleeping position	SDNN, SDNNi, SDANNi, RMSSD, pNN50, VLF*, LF*, HF*	computerised polygraph recording system (Morpheus system, Medatec, Belgium)	included: normal medical history, normal clinical and neurological examinations, normal ECG patterns for conceptional age, polygraphic results without cardiac or respiratory abnormalities, sinus rhythm on a surface ECG	age, heart rate, breathing rate	1000	mean ± SD	19
Mehta et al. 2002	asymptomatic infants	96 (50 m, 46 f)	24 - 72 h	24h ECG monitoring; analyzed data ranged from 2.37-23.76 h, median 18.5±4.9 h; 8 recordings had a recording time of <12 h	SDNN, SDANN, SDNNi, RMSSD, NN50, TP, ULF, VLF, LF, HF, HRV triangular index	3-channel Holter monitor (Marquette M-8500, Milwaukee, Wisconsin)	>2400g, gestational period 34-42 weeks; excluded: mothers who took medication during pregnancy known to affect cardiovascular system, mothers with acute or chronic diseases (hypertension, DM), symptomatic or required oxygen for <5 mins, ventilatory support, ICU-support, children with congenital anomalies; 2-dimensional ECG and Doppler examinations in each children	Heart rate	125	mean ± SD and percentile (5th und 95th)	18

Study	Cohort	n	Age [years]	Measurement interval, time of day if short-time measurement	HRV parameters	Device	Confounders considered	References values presented for	Sampling rate in Hz	Norm values presented as…	STARD-HRV
Michels et al. 2013	healthy children from Belgium (Aalter)	460 (240 m, 220 f)	5 - 10	9am – 6pm, 10 minutes examination, quiet room, supine position, normal breathing; middle 5 minutes manually checked for analyzing, or if necessary, another 5-min interval was selected	meanRR, SDNN, RMSSD, pNN50, VLF, LF, HF, LF/HF	Polar Wearlink 31 chest belt with Windlink infrared computer transmitter	cardiovascular disease	age, gender, time point, physical activity (accelerometry), physical fitness (cardiopulmonary fitness, upper and lower limbmuscular fitness) and body composition (BMI, fat%, fat and fat-free mass)	1000	median (IQR) and percentile (2.5th, 25th, 50th, 75th, 97.5th)	20
O'Neal et al. 2016	multi-ethnic subjects from North-American hospitals/ field centers	1175	45 – 84	3 consecutive 10 second ECGs	SDNN, RMSSD	12 channel ECG (GE MAC 1200)	Excluded: reported smoking, CVD, CVD risk factors, DM, hypertension, dyslipidemia, obesity, major ECG abnormalities as defined by Minnesota code classification	gender, ethnicity	n/a	mean ± SD and percentile (2th und 5th)	18
Otsuka et al. 1999	subjects in clinical health	141 m	m 7 - 92, f 3 – 86	1-hour intervals for 24h, some parameters also analysed for 1 or 5 min	SDNN, RMSSD, NN50, pNN50, SDANN,SDmean, LF, HF, TF, LF/HF	not specified	n/a	age, gender, daytime, circadian variation	n/a	mean ± SD	12
Park et al. 2007	healthy subjects	637 (366 m, 271 f)	45.1 ± 10.7	5 min, resting, after an overnight fast	SDNN, RMSSD, TP*, LF*, HF*, LF/HF*	SA-2000E, Medicore, Korea	rountine laboratory and physical examinations to eliminate cardiovascular and neurological disorders; excluded: significant artefacts, ischemic heart disease, bradycardia/ tachycardia	age, gender, heart rate, hypertension, DM, obesity	500	mean ± SD	14

**Study**	**Cohort**	**n**	**Age [years]**	**Measurement interval, time of day if short-time measurement**	**HRV parameters**	**Device**	**Confounders considered**	**References values presented for**	**Sampling rate in Hz**	**Norm values presented as…**	**STARD-HRV**
Patural et al. 2019	full term newborns from Saint Etienne, France	271	0 - 24 months	24h, either from polysomnographic recordings or ECG Holter	meanRR, meanHR, pcNN20, pcNN30, pcNN50, RMSSD, SDANN, SDNNi, HRC triangular index, TINN, X, Y, M, N, TP, ULF, VLF, LF, HF*, LF/HF*, Centroid, SD1, SD2, SD1/SD2, SD1nu, SD2nu, pLF1, pLF2, pHF1, pHF2, IMAI1, IMAI2 (ratio between low and high frequency indices), DFA1, DFA2, H DFA, H Higuchi, H Katz, Hurst (the last 3 being measures of the self-similarity of the RR signal), Beta 1/f slope (calculated on the PSD plotted on a log-log scale from 10^-4 to 10^-2 Hz), Skewness, Kurtosis, LE, Acceleration Capacity (AC), Deceleration Capacity (DC), AppEn, SampEn, ShanEn, Conditional Entropy (CE), corrected CE (CCE), normalized CCE, rho (Entropy), Lempel-Ziv Complexity	0 and 6 months old → polysomnographic recording (Dream Medatec, Belgium)<br><br>12-24 months old → 24h ambulatory ECG (Vista, Novacor, Rueil Malmaison, France)	n/a	age, gender	200; ECG resampled at 1000	percentile (3th, 10th, 50th, 90th, 97th)	15

Study	Cohort	n	Age [years]	Measurement interval, time of day if short-time measurement	HRV parameters	Device	Confounders considered	References values presented for	Sampling rate in Hz	Norm values presented as…	STARD-HRV
Piccirillo et al. 1995	healthy volunteers	83 (42 m, 41 f)	25 - 85 (divided into three age groups: <44, 44 - 64, >64)	30 min supine rest, then 15 mins ECG recording; then head up tilt with tilt table, after 15 mins 90°upright, back to supine position (0°) (transit time about 15 seconds) and another 15 min ECG measurement; If systolic BP changed by >20 mmHg, stop of testing and exclusion of the participant	VLF, LF, HF, LF/HF, TP, CF (center frequency) as natural logarithms and normalized units, power spectral calculated from a consecutive series of 512 RR intervals	ECG	exclusion; diastolic BP ≥95 mmHg; systolic BP ≥160 mmHg; BMI > 26 kg/m²; smoking (> 5 cigarettes per day); DM (presence of glucosuria or fasting glycemia > 120 mg/dl or 110 mg/dl at 2 h after glucose loading); cholesterolemia ≥ 220 mg/dl; and history or demonstrable evidence of cardiovascular, respiratory, renal (presence of proteinuria and creatinine > 1.5 mg/dl); hepatic, gastro-intestinal, or systemic disease. Complete history obtained, full clinical examination followed by resting ECG and ECG under stress, 2D-echo Doppler study of neck vessels; ECG with >1% ectopic beats excluded as well	age	500	Mean ± SD and 95%-KI for some parameters	16

Study	Cohort	n	Age [years]	Measurement interval, time of day if short-time measurement	HRV parameters	Device	Confounders considered	References values presented for	Sampling rate in Hz	Norm values presented as…	STARD-HRV
Pikkujämsä et al. 2001	healthy middle-aged subjects from the Oulu district, Finland	389 (189 m, 200 f)	40 - 59 (m 50 ± 6; f 51 ± 6)	ECG in supine position for 6 min (calibration of ECG recorder), then 15 mins measuring in quite supine position, 15 mins sitting, 15 mins while walking; for better standardisation, middle 13 mins of supine and sitting periods were used for analysis	MeanRR, SDNN, LF, HF, LF/HF, DFA1, ApEn	ambulatory ECG recorder	excluded: entitled to a refund of antihypertensive medication, angina pectoris, dyspnea, ECG or echocardiographic evidence of heart disease, cardiovascular medication, DM or fasting blood glucose >6.7 mmol/l, elevated BP during ambulatory BP recording (>140/90 mmHg), technical artifacts or rhythm abnormalities during ECG. Interview and standardized questionnaire for past medical history, medication, cardiac symptoms, smoking habits, alcohol consumption, physical activity, personality type; clinical examination; laboratory tests (e.g. 2h-glucose-tolerance-test)	gender, standing/ sitting position (SDNN, LF, HF)	n/a	mean ± SD and range	14
Ramaekers et al. 1998	healthy subjects	276 (141 m, 135 f)	18 - 71	24h between 8am and 9am; 8am-9pm, 23pm-6am	RMSSD, SDNN, SDANN, pNN50, TP*, LF, LFnu, HF, HFnu, LF/HF	Holter recorder	detailed medical history obtained<br>Excluded: DM, hypertension, cardiovascular, neurological or psychiatric diseases	(age), gender, day/ night changes	200	mean ± SD and percentile/KI (5th - 95 th)	14
Saleem et al. 2012	healthy individuals	45 (27 m, 18 f)	29 - 80 (42 ± 14)	24h	SDNN, SDANN, SDNNi, RMSSD, pNN50, TP, VLF, LF, HF	12-chanel Holter ECG (Motara ELI 250 EKG Machine; Absolute Medical Equipment, New York)	normal physical examination and ECG; excluded: DM, cardiovascular, neurological or psychiatric diseases	gender	n/a	mean ± SD	13
Sammito et al. 2016	voluntary subjects	695 (319 m, 376 f)	20 - 60	24h	SDNN, RMSSD, SDANN, pNN50, LFnu, HFnu, LF/HF, SD1, SD2	2-channel ECG (Schiller MT101, Firma Schiller AG, Baar, Switzerland)	excluded: diagnosis of coronary heart disease with or without stent implantation, heart attacks, strokes, night shifts during recording time, diagnosed DM (irrespective of type), use of psychiatric medication, pregnancy	age, gender	1000	percentile (5th, 25th, 50th, 75th, 95th)	18

Study	Cohort	n	Age [years]	Measurement interval, time of day if short-time measurement	HRV parameters	Device	Confounders considered	References values presented for	Sampling rate in Hz	Norm values presented as…	STARD-HRV
Sammito et al. 2017	voluntary subjects	673 (301 m, 372 f)	20 - 60	24h measurement, 5 minutes (2am – 02.05am) for HRV-analysis	SDNN, RMSSD, pNN50, HRTV tri. Index, TINN, LF/HF, LFnu, HFnu	2-channel ECG (Schiller MT101, Firma Schiller AG, Baar, Switzerland)	excluded: diagnosis of coronary heart disease with or without stent implantation, heart attacks, strokes, night shifts during recording time, diagnosed DM (irrespective of type), use of psychiatric medication, pregnancy	age, gender	1000	percentile (5th, 10th, 25th, 50th, 75th, 90th, 95th)	18
Seppala et al. 2014	mainly prepubertal children from Kuopio, Finland	465 (239 m, 226 f)	6 - 8	Between 9am and 12 pm, 1h after breakfast, 10 minutes resting still in supine position, then 5 minutes of ECG recording lying still on bed, 1- and 5-minutes samples selected for HRV-analysis	meanRR, meanHR, SDNN, RMSSD, pNN50, TINN tri.index, TINN, LF, HF, LFpeak, HFpeak, LF/HF, SD1, SD2, SD1/SD2, SampEn, D2, DFA1, DFA2	12-channel ECG (Cardiosoft V6.5 Diagnostic System, GE Healthcare Medical Systems, Freiburg, Germany)	n/a	age, gender, maturity stage, height, weight, BMI-SDS (standard deviation score)	500	percentile (5th, 25th, 50th, 75th, 95th)	18
Silvetti et al. 2001	healthy children and adolescents	103 (57 m, 46 f)	1 – 20	24h, day and night cycle, normal daily activity	meanHR, SDNN, SDNNi, SDANN, RMSSD, pNN50	ECG Holter Monitoring (MR45 and MR45-3 oxford recorder)	underwent physical examination, standard ECG, Doppler, Holter monitoring, exercise test; No drugs, no competitive sports, no history of syncope, no significant artefacts, >200 isolated premature atrial or ventricular beats, pause >2s, any AV-block	age, gender	n/a	mean ± SD	12
Sitovskyi et al. 2020	healthy adolescents & school-children	812 (388 m, 424 f)	12 – 13	5 min ECG after 10 mins resting	meanRR, SDNN, RMSSD, pNN50, CVRR, VLF, %VLF, LF, %LF, LFnu, HF, %HF, HFnu, TP, IT arb.u. (index of tension)	Poly-Spectrum-12 complex (Neurosoft, Russia)	n/a	age, gender	n/a	median, percentile (25th, 75th)	11
Sloan et al. 2008	Young adults	757 (321 m, 436 f)	33 – 47 (40.0 ± 3.7)	quiet rest in seating position for 2 mins, then 10 mins data collection	RRV computed as the mean from 2 5 min-epochs (10min split in half) LF, HF*, LF/HF, SDRR	no information	light breakfast, no smoking or caffeinated beverages before	age, gender, ethnicity	500	mean ± SD	17
Sosnowski et al. 2002	healthy subjects	296 (215 m, 81 f)	47 ± 10	24h	meanRR, SDNN, SDANN, HRV fraction (HRVF)	Ambulatory ECG	no signs or symptoms of cardiovascular disease in physical examination, normal 12 lead ECG, no drugs	age	n/a	mean ± SD and percentile (2.5th, 97.5th)	10

Study	Cohort	n	Age [years]	Measurement interval, time of day if short-time measurement	HRV parameters	Device	Confounders considered	References values presented for	Sampling rate in Hz	Norm values presented as…	STARD-HRV
Tang et al. 2014	subjects from rural and urban Shanghai	371 (78 m, 293 f)	30 – 80 (56.5 ± 8.75)	15 min ECG	TP, LF, HF, LFn, HFn	ECG (HMX-3C electrosphygmo graph transducer)	clinically stable condition, no previous medical history of DM, hypertension, dyslipidemia, coronary artery disease, cerebral stroke or heart failure; fasting plasma glucose (FPG) <100 mg/dL, 2 h plasma glucose (2hPG) <140 mg/dL after 75 g oral glucose tolerance test; normal BMI 18.5 - 24.9 kg/m²; triglycerides <150 mg/dL, high-density lipoprotein cholesterol >40 mg/dL; systolic BP <140 mmHg, diastolic BP <90 mmHg; Excluded: use of any medications that may affect resting HR, e.g. β-receptor blockers, 1 month before the study	age	n/a	mean ± SD and (not in total) percentile (2.5th, 5th, 10th, 50th, 97.5th)	16
Tegegne et al. 2020	cohort study, North of the Netherlands	84.772 (34.333 m, 50.439 f)	13 - 91 (40.8)	10 seconds, resting	RMSSD, $RMSSD_c$	ECG	n/a	age, gender	n/a	mean ± SD, percentile (2nd, 98th) and centile curves	9
Tsuji et al. 1996	subjects from Framingham, UK	1918 (865 m, 1053 f)	20 - 79 (50 ± 13)	first 2h of ambulatory ECG recordings >60 min analyzable data, at least 50% time processed, premature beats <10% of total beats	SDNN, RMSSD, pNN50, VLF*, LF, HF, LF/HF	Holter ECH (Cardiodata/Mortara Mk5)	routine examination; excluded: transient or persistent nonsinus rhythm, premature beats >10% of total beats, <1h recorded time or processed time <50% of recorded time, antiarrhythmic medications, heart rate for reference value group must be 40-99/min	age, heart rate	32	mean ± SD	15

**Study**	**Cohort**	**n**	**Age [years]**	**Measurement interval, time of day if short-time measurement**	**HRV parameters**	**Device**	**Confounders considered**	**References values presented for**	**Sampling rate in Hz**	**Norm values presented as…**	**STARD-HRV**
Umetani et al. 1998	healthy subjects from the US	260 (112 m, 148 f)	10 – 99	24h	SDNN, SDNNi, SDANN, RMSSD, pNN50	179 with Cardionostics Dura-Lite recorder (Cardionostics) 81 with Del Mar 459 recorder 3 different analysing programs used "randomly selected Holter tapes" analyzed with all 3 programs	healthy subjects without clinical evidence of organic disease in terms of medical history, physical examination, rest 12-lead electrocardiogram, routine blood chemistry profiles and complete blood count; no medication except for oral contraceptives and non-steroidal inflammatory agents; excluded: recording duration <20h or >10% nonsinus beats; high frequency or high grade supraventricular ectopic beats	age, gender	n/a	mean ± SD and lower and upper 95% confidence limits	14
Urooj et al. 2011	healthy male Indian subjects	100 m	18 - 45 (17.6 ± 6.7)	30 min	$SDRR_{Magid}$, $SDRR_{Kleiger}$, RMSSD, pNN50	ECG (Mortara Portrait Electrocardiograph Holter H-12 Recorder)	study subjects judged to be medically healthy based on demographic data (including age, sex, history of smoking and alcohol consumption, body weight and height), vital signs, physical examination, ECG, chest X-ray, clinical laboratory tests (including disease markers of syphilis, HIV, hepatitis B and C), urinary drug screening; brief clinical examination (cardiovascular and respiratory system, abdominal, CNS). Excluded: smoker, borderline hypertension		n/a	mean ± SEM	14

Study	Cohort	n	Age [years]	Measurement interval, time of day if short-time measurement	HRV parameters	Device	Confounders considered	References values presented for	Sampling rate in Hz	Norm values presented as…	STARD-HRV
Van den Berg et al. 2018	subjects from the Netherlands	13.943 (5.889 m, 8.054 f)	11 days - 91 years	10 seconds, supine position	$SDNN_c$, $RMSSD_c$	Five different 12-channel ECG (1. Cardio Control, Delft, Netherlands; 2. Megacart electrocardiographs, Siemens, Erlangen, Germany; 3. CardioPerfect equipment, Welch Allyn Cardio Control, United States; 4. CardioPerfect equipment, Welch Allyn Cardio Control, United States; 5. ACTA electrocardiograph; Esaote, Florence, Italy)	excluded: history of myocardial infarction, heart failure, coronary bypass surgery, coronary angioplasty, pacemaker implantation, hypertension, DM, use of antihypertensive medication (including beta-blockers); excluded for ECG abnormalities: excessive noise, excessive baseline wander, sudden baseline jumps, spikes, premature ventricular beats, premature supraventricular beats, second- or third-degree atrioventricular block	age, gender, heart rate	500-1200 (higher for the pediatric group)	mean, percentile (2nd, 98th) and centile curves	15
Voss et al. 2012	healthy subjects from Bavaria, Germany	1906 (1124 m, 782 f)	25 – 74	5 min, resting, supine position	meanRR, SDNN, CVRR, SDANN1, RMSSD, pNN50, pNNI20, renyi4, wsdvar, a21rr, shannon_h, LF, HF, P, LF/HF, LF/P, HF/P, LFn, HFn, shannon_SD, forbword, wpsum02, wpsum13, , phvar5, plvar20, fwrenyi025, fwrenyi4, DFA1, DFA2, $Hc^{3,3}$, ST_MP, ST_0V, ST_1V, ST_2V, ST_INC, ST_DESC, ST_PEAK, ST_VAL, ST_2LV, ST_2UV, SD1, SD2, SD1/SD2, SPPA_c_4, SPPA_c_5, SPPA_c_6, SPPA_c_7, SPPA_c_8, SPPA_c_9, SPPA_r_4, SPPA_r_5, SPPA_r_6, SPPA_r_7, SPPA_r_8, SPPA_r_9, SPPA_entropy, AS1, AS2, AS3, , a31rr, a21rrcor, a31rrcor, x2peakrrcor, y2peakrrcor, amax21rrcor	ECG, lead II and lead V2 simultaneously	subjects excluded for documented diseases, intake of medication possibly affecting heart rate regulation or established cardiac arrhythmia	age	500	median (ICR) (25th - 75th)	15

Study	Cohort	n	Age [years]	Measurement interval, time of day if short-time measurement	HRV parameters	Device	Confounders considered	References values presented for	Sampling rate in Hz	Norm values presented as…	STARD-HRV
Ziegler et al. 1992	healthy subjects	120 (40 m, 80 f)	15 – 67 (35.2 ± 11.8)	in the morning; at least 5 min rest, then 5 min measurement of HRV in supine and standing position: HR, CV and RMSSD calculated from 150 RR-intervals free of artefacts; with breathing: supine rest, instruction to deeply breath at 6 cycles/ min; HR, CV and RMSSD calculated from 100 RR-intervals without artefacts	RMSSD, $RMSSD_b$, CVRR, LF, MF, HF	ProSciCard system (MediSyst, Linden, Germany)	excluded: history of diseases of the cardiovascular system, any concurrent illness, medication know to influence cardiac, vascular or autonomic function	age	1000	percentile (2.3th)	19
Ziegler et al. 1999	healthy subjects	94 (40 m, 54 f)	22 - 73 (40.5)	24h 2-channel ECG; split into 5 min segments for analysis, mean of 12 = 1h HRV-values; 8 out of 12 segments to be analyzed as 1h, else missing; >18h for 24h measure, >12h for day period (6am - midnight), >4h for night period (midnight - 6am)	SDNN-Index, CVRR, NN50, RMSSD, triangular index, TINN, top angle index (TAI), VLF, LF, HF, LF/HF, LFn	Cardiolight system (Medset, Hamburg, Germany)	n/a	age, gender, time day/night	200	SD percentile (2.5th)	16

* analyzed frequency parameters with other frequency bands as in guidelines (Task Force of The European Society of Cardiology and The North American Society of Pacing and Electrophysiology, 1996; Sammito et al. 2024) (VLF 0.003 – 0.04 Hz, LF 0.04 – 0.15 Hz, HF 0.15 – 0.4 Hz)

b = breathing

BMI = Body-Mass-Index

BP = blood pressure

c = corrected for heart rate

COPD = chronic obstructive pulmonary disease

CVD = cardiovascular disease

DM = Diabetes mellitus

f = female study participants

IQR = Interquartilrange

LLE = largest Lyapunov exponent

m = male study participants

SD = standard deviation

SEM = standard error of the mean
